# GKLOMLI: a link prediction model for inferring miRNA–lncRNA interactions by using Gaussian kernel-based method on network profile and linear optimization algorithm

**DOI:** 10.1186/s12859-023-05309-w

**Published:** 2023-05-08

**Authors:** Leon Wong, Lei Wang, Zhu-Hong You, Chang-An Yuan, Yu-An Huang, Mei-Yuan Cao

**Affiliations:** 1 Guangxi Key Lab of Human-machine Interaction and Intelligent Decision, Guangxi Academy of Sciences, Nanning, 530007 China; 2 College of Information Science and Engineering, Zaozhuang University, Zaozhuang, 277160 China; 3 School of Computer Science, Northwestern Polytechnical University, Xi’an, 710139 China; 4 School of Electrical and Electronic Engineering, Guangdong Technology College, Zhaoqing, 526100 China; 5 Faculty of Information Science and Technology, Universiti Kebangsaan Malaysia, UKM, 43600 Bangi, Selangor Malaysia; 6 Institute of Machine Learning and Systems Biology, School of Electronics and Information Engineering, Tongji University, 200092 Shanghai, China

**Keywords:** Computational biology, miRNA–lncRNA interaction, Link prediction, Competing endogenous RNA (ceRNA), Gaussian kernel

## Abstract

**Background:**

The limited knowledge of miRNA–lncRNA interactions is considered as an obstruction of revealing the regulatory mechanism. Accumulating evidence on *Human* diseases indicates that the modulation of gene expression has a great relationship with the interactions between miRNAs and lncRNAs. However, such interaction validation via crosslinking-immunoprecipitation and high-throughput sequencing (CLIP-seq) experiments that inevitably costs too much money and time but with unsatisfactory results. Therefore, more and more computational prediction tools have been developed to offer many reliable candidates for a better design of further bio-experiments.

**Methods:**

In this work, we proposed a novel link prediction model based on Gaussian kernel-based method and linear optimization algorithm for inferring miRNA–lncRNA interactions (GKLOMLI). Given an observed miRNA–lncRNA interaction network, the Gaussian kernel-based method was employed to output two similarity matrixes of miRNAs and lncRNAs. Based on the integrated matrix combined with similarity matrixes and the observed interaction network, a linear optimization-based link prediction model was trained for inferring miRNA–lncRNA interactions.

**Results:**

To evaluate the performance of our proposed method, *k*-fold cross-validation (CV) and leave-one-out CV were implemented, in which each CV experiment was carried out 100 times on a training set generated randomly. The high area under the curves (AUCs) at 0.8623 ± 0.0027 (2-fold CV), 0.9053 ± 0.0017 (5-fold CV), 0.9151 ± 0.0013 (10-fold CV), and 0.9236 (LOO-CV), illustrated the precision and reliability of our proposed method.

**Conclusion:**

GKLOMLI with high performance is anticipated to be used to reveal underlying interactions between miRNA and their target lncRNAs, and deciphers the potential mechanisms of the complex diseases.

## Introduction

In pre-genomic eras, the genetic central dogma plays a vital role in deciphering the message flow of genetic material [1]. However, along with the in-depth studies, it was found that the biological mechanism is far more complex than the dogma [2, 3]. From the recent researches, more and more non-coding RNA (ncRNA) that cannot directly translate into protein has been found that it can function as regulation in most of the biological processes [4]. The ncRNA makes up more than 98% of total RNA. There are many criteria for ncRNA classification. According to the length of RNA and the significance, microRNA (miRNA) with 20–25 nt and long non-coding RNA (lncRNA) with > 200 nt are two main kinds of ncRNA. miRNA can combine with the Argonaute proteins and function as the RNA-induced silencing complex (RISC), which leads to target mRNA degradation and translation repression [5, 6]. lncRNA has similarity with protein-coding RNA in splicing structure and length [7]. The competing endogenous RNA (ceRNA) hypothesis indicates that crosstalk among RNAs is existing, which results in various RNA regulation in vivo [3, 8].

The research on miRNA–target interaction (MTI) is a hot topic for its regulatory role in proliferation and apoptosis, cell differentiation, cellular transport, transcriptional and post-transcriptional regulation, epigenetic regulation, cell cycle control, tumorigenesis, and organ or tissue development [9–11]. It is known that some specific miRNAs can act as response elements, also called MREs. Gene expression can be inhabited when RNAs need to bind with the same MREs competitively [12]. It is reported that miRNAs can regulate lncRNAs for which some specific lncRNAs have similar structures with mRNA [13]. However, more and more studies release that miRNA–lncRNA interactions (MLIs) play essential roles in human diseases such as tumors, cancer, and vasculature [14–16]. It is believed that more effective therapeutic approaches can be developed by further investigating the regulation activities among miRNAs and lncRNAs [10, 13].

To reveal the underlying mechanisms of regulatory mediated by miRNAs and lncRNAs, the interactions between them need to be validated [11]. Owing to the next-generation bioengineering techniques, it is conducive to help researchers to carry out the bio-experiments in high throughput, obtain the results, and build a normative database [17, 18]. Based on the cumulative bio-data, the researchers in other fields can easily get access and make more efforts for further investigation.

The experimental identification method can intuitively detect the functions of miRNAs acting on their targets. Many well-established online databases collect miRNA-related records with details, and update periodically, such as miR2disease, miRCancer, OncomiRDB, DIANA-TarBase, microRNA.org, miRGate, miRDB, miRNAMap and human microRNA disease database (HMDD) [19–27]. By using the database management techniques and web-based techniques, these records can be freely accessed and browsed through multiple filters. For instance, DIANA-TarBase v8.0 collects more than 665,800 miRNA interactomes with details about publication, cell lines, tissue, and experiments, as well as implements an intuitive interface with searching filters including species, cell type, tissue, regulation type, and other options. Therefore, accumulating bio-data facilitates researchers to make further progress.

Biogenetical deregulation of some specific miRNAs can lead to various human diseases such as miR-17-92 at malignant lymphoma [28], miR-206 at sensing motor neuron [29, 30], and miR-1 at cardio-genesis [31]. However, the validation progress of miRNA interactomes is still underway. The regulation functions of most miRNAs are far from clear, for which it may be affected by the evolution of species, gene mutation, dynamic in vivo, and other uncertain factors [32].

To date, an optimal framework for revealing mechanisms of ncRNA-target duplexes is to integrate experimental and computational approaches iteratively [33, 34]. By using the limited understanding of miRNAs and the related targets, computational methods can be developed to predict potential interactions that have high probabilities to be true positive ones in further biological experiments. Many existing computational prediction tools are combined with some wide-used principles such as evolutionary conservation status, seed sequence complementarity, target-site abundance, target-site accessibility, free energy, G-U wobble, and local AU flanking content [5]. There are many miRNA–target prediction tools based on different combination of principles that can improve the performance of prediction to a certain extent.

Specifically, LncTar was proposed to predict the RNA target of lncRNA by integrating the primer-dimer prediction method implemented by PerlPrimer, and normalized free energy that can measure the relative stability of base pairing between RNAs [35]. A pattern-based approach rna22 was proposed to identify miRNAs and their corresponding heteroduplex, which facilitates users to obtain prediction results with different binding sites that are recognized by using Teiresias algorithm [36]. A parameter-free prediction model, Probability of Interaction by Target Accessibility (PITA), was proposed to apply target-site accessibility principle to detect miRNA–target duplexes according to the energy change in procedure of RNAs' formation and unpairing [37]. miRanda was proposed to predict the miRNA:target based on evolutionary conservation, seed sequence complementarity, G-U wobble and free energy [38]. TargetScan was developed based on multiple principles such as local AU content, seed sequence complementarity, target-site accessibility, target-site abundance, evolutionary conservation, G-U wobble, compensatory pairing and free energy [39]. Moreover, some web-based prediction tools with powerful computing are developed to facilitate researchers to dispose large-scale data, such as STarMirDB, DIANA-microsT-CDS, miRGate and miRDB. Although these existing tools were well-established, many efforts still need to be made on the researches of miRNA:target.

Most of the tools mentioned above rigidly relied on the conserved seed match, which may yield a high false-negative rate because of the sophistication of the mechanism in vivo [40–45]. In recent years, many existing in silico prediction methods of miRNA:target were developed based on machine learning (ML) algorithms. ML algorithms can make it possible to do the prediction task more applicable and effective for various species and miRNA-related targets by learning the most potential features from the limited experimental data. Nevertheless, more and more flexible methods were developed to predict miRNA:target by constructing an effective interaction network with more entities and combining the side information.

Due to the vital role of MLI, some specific prediction algorithms have been proposed for their particularity. Yu et al*.* developed a resource allocation-based algorithm called LCBNI that integrated the similarities based on the sequence profile [46]. LNRLMI was proposed to use the expression profile of RNAs to construct the RNA similarities, in which the co-expression information among RNAs can be utilized for model training through the integrated network [47]. LMNLMI is a matrix completion model combined with multiple profiles for prediction [48]. INLMI and EPLMI are both based on a two-way diffusion [49, 50]. The difference is that INLMI computed the final rating scores by taking the average of the results from the sequence-based and expression profile-based model, and EPLMI trained two sub-models where one is based on miRNA expression profile-related weighting network and one is based on lncRNA expression profile-related weighting network.

In this work, motivated by the previous works, we here propose a linear optimization-based method combined with Gaussian kernel-based network similarity for inferring miRNA–lncRNA interactions. In detail, A Gaussian kernel-based method was employed to construct the similarity on the given observed miRNA–lncRNA interaction network. Then, the training matrix was constructed by combining the interaction network and the similarities of miRNAs and lncRNAs. Finally, a link prediction model was trained by using the well-constructed training matrix. For performance evaluation, *k*-fold cross-validation (CV) experiments were implemented by setting *k* to 2, 5, and 10. Also, leave-one-out cross-validation (LOO-CV) was carried out. Specifically, by carrying out each *k*-fold CV with a randomly given interaction profile 100 times, our proposed method yielded high AUCs at 0.8623 ± 0.0027 (2-fold CV), 0.9053 ± 0.0017 (5-fold CV), 0.9151 ± 0.0013 (10-fold CV), and 0.9236 (LOO-CV). From the results yielded by the existing methods and our proposed method, it is believed that our proposed method can yield reliable results for obtaining more potential miRNA–lncRNA interactions and uncover the underlying regulatory mechanisms.

## Results

### Using *k-*fold* cross-validation* for performance evaluation

For the purpose of performance evaluation, it is universal to apply the *k-*fold cross-validations (CV) method that can help to measure more precisely to a certain extent. In this paper, 4 kinds of CV experiments were implemented, including 2-fold CV, 5-fold CV, 10-fold CV, and leave-one-out CV (LOO-CV). A smaller value of *k* indicates the less training samples. Before training model, all samples are shuffled and divided into *k* parts equally. *k* − 1 parts are for training and the rest for testing. When employing 5-fold CV, 5118 samples of MLIs used in our work are divided into 4095 samples for training and 1023 samples for testing. The 5-fold CV is widely used for comparison among models.

**Fig. 1:**
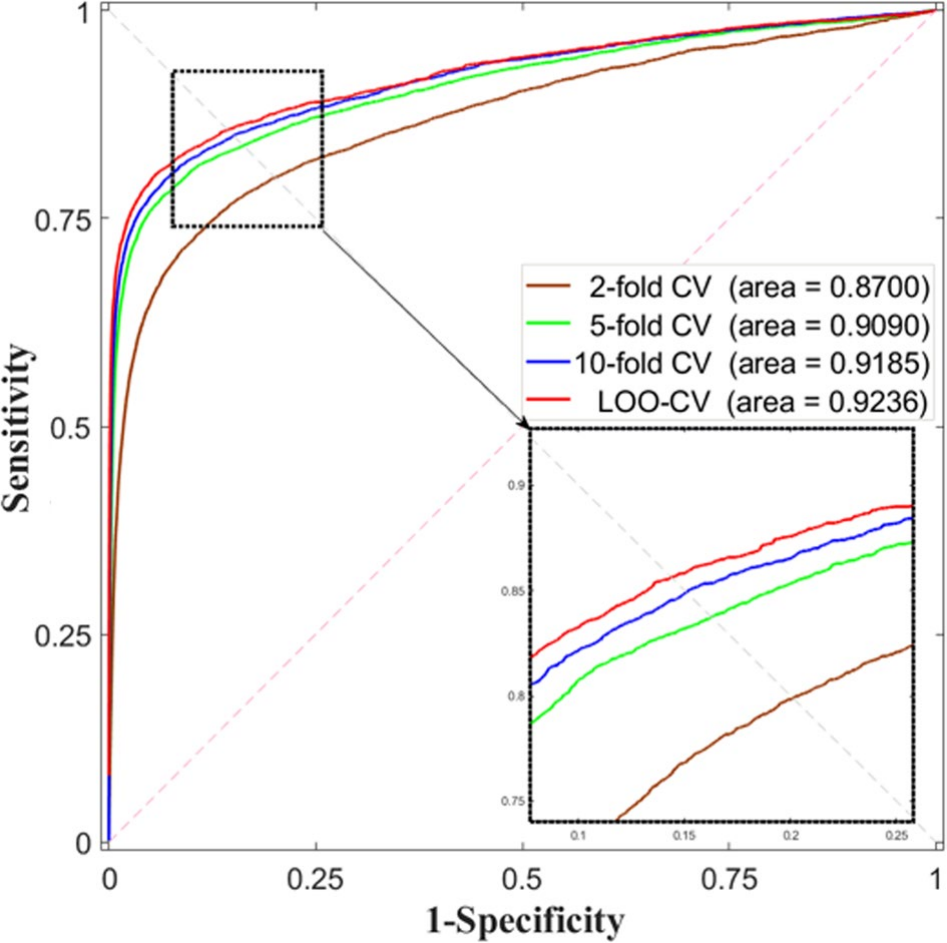
ROC curves of GKLOMLI by implementing 2-fold, 5-fold, 10-fold CV and LOO-CV

On account of that training model with different training sets yield different performance, each *k*-fold CV is implemented for 100 times. 2-fold, 10-fold CV and LOO-CV were implemented and the results are listed in Table 1. As a result, our proposed method that is integrated with Gaussian kernel-based network similarity yielded the average AUCs at 0.9053 and a low standard deviation at 0.0017 in 5-fold CV. Also, the AUCs are at 0.8623 ± 0.0027 (2-fold CV), 0.9151 ± 0.0013 (10-fold CV), and 0.9236 (LOO-CV). To better illustrate the performance, all the receiver operation characteristics (ROC) curves were plotted in Fig. 1.

**Table 1:** Performance comparison by implementing CV experiments on different profiles

Profile	2-fold CV	5-fold CV	10-fold CV	LOO-CV
No profile	0.8306 ± 0.0037	0.8806 ± 0.0020	0.8910 ± 0.0014	0.8997
Expression	0.8378 ± 0.0033	0.8903 ± 0.0021	0.9017 ± 0.0014	0.9112
Bio-function	0.8389 ± 0.0031	0.8890 ± 0.0022	0.9008 ± 0.0018	0.9111
Sequence	0.8515 ± 0.0031	0.8941 ± 0.0020	0.9040 ± 0.0016	0.9123
GKLOMLI	0.8623 ± 0.0027	0.9053 ± 0.0017	0.9151 ± 0.0013	0.9236

### Experiment results in different types of RNA profile-based similarity

**Fig. 2:**
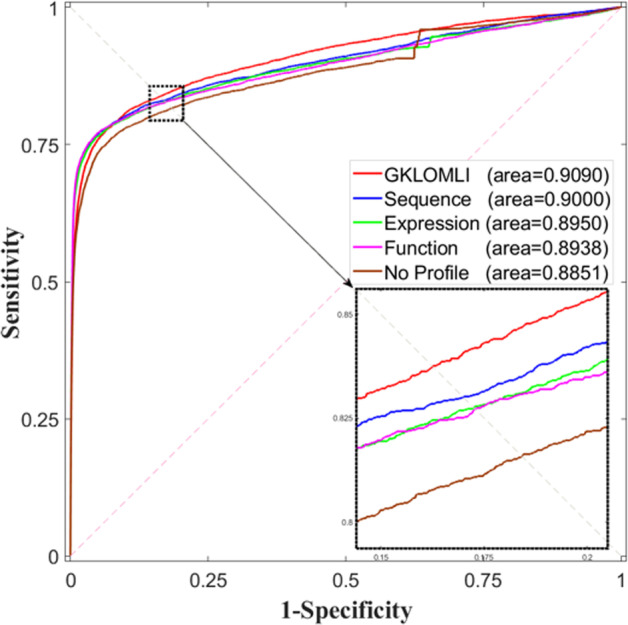
ROC curves by implementing 5-fold CV on different profiles and no profile

To demonstrate the outstanding performance of our proposed method, we here implement *k*-fold CV with the other three types of profiles and without profile. The results are listed in Table 1. The corresponding ROC curves were plotted in Fig. 2. From the results of 2-fold CV, the AUCs are at 0.8306 ± 0.0037 (no profile), 0.8378 ± 0.0033 (expression profile), 0.8389 ± 0.0031 (bio-function), and 0.8515 ± 0.0031 (sequence), respectively. From the results of 5-fold CV, the AUCs are at 0.8806 ± 0.0020 (no profile), 0.8903 ± 0.0021 (expression profile), 0.8890 ± 0.0022 (bio-function), and 0.8515 ± 0.0031 (sequence), respectively. Also, *p* values between our proposed method and the methods with the other three types of profiles and without profile are calculated at 3.4646e−154 (no profile), 1.6730e−95 (expression profile), 1.0972e−107 (bio-function) and 3.7514e−93 (sequence), respectively. From the results of 10-fold CV, the AUCs are at 0.8910 ± 0.0014 (no profile), 0.8903 ± 0.0021 (expression profile), 0.9008 ± 0.0018 (bio-function), and 0.9040 ± 0.0016 (sequence), respectively. From the results of LOO-CV, the AUCs are at 0.8997 (no profile), 0.9112 (expression profile), 0.9008 ± 0.0018 (bio-function), and 0.9123 (sequence), respectively.

From these results, the model without any profile yields the lowest AUC overall. From the results of the models based on different bio-profiles, sequence-based model performs well. It should be noticed that the collection of bio-profiles are incomplete except the sequence information, which indicated that the lack of information tends to lead to a low AUC. From the 5-fold results, the *p* values are lower than 0.05, which indicates our method has better performance than those methods.

### Comparison with different prediction methods

To better illustrate the good performance of our proposed method, the current widely used prediction methods were introduced for comparison, including LCBNI [46], LNRLMI [47], LMNLMI [48], INLMI [50], and EPLMI [49]. Yu et al. proposed a method named LCBNI to predict interactions between miRNAs and lncRNAs by employing a resource allocation algorithm combined with the sequence-based similarity of RNAs. LNRLMI method was proposed to apply the co-expression mechanism in a miRNA–lncRNA interaction network to construct the expression profile-based similarity and integrate that for prediction. LMNLMI method was first proposed to integrate three types of biological profiles relating to biological function, sequence information, and expression. INLMI employed a non-negative matrix factorization method on an integrated network utilizing interaction profile, sequence information-based, and expression profile-based similarities. EPLMI was first proposed to predict miRNA–lncRNA interactions by using a two-way diffusion model based on the expression profiles of miRNA and lncRNA.

From the 5-fold CV experiment results listed in Table 2, LNRLMI and EPLMI achieved the average AUCs at 0.8960 and 0.8447, respectively. And, LCBNI, LMNLMI and INLMI achieved the best AUCs at 0.8982, 0.8926 and 0.8517, respectively. Our proposed method yielded the best AUC at 0.9090 and the average AUC at 0.9053. The outperformance of our proposed method indicated that it can be a better method for inferring miRNA–lncRNA interactions.

**Table 2:** Performance comparison of different existing methods

Method	LCBNI [46]	LNRLMI [47]	LMNLMI [48]	INLMI [50]	EPLMI [49]	GKLOMLI
AUC	0.8982	0.8960 ± 0.0015	0.8926	0.8517	0.8447 ± 0.0017	0.9053 ± 0.0017

### Parameter selection

**Fig. 3:**
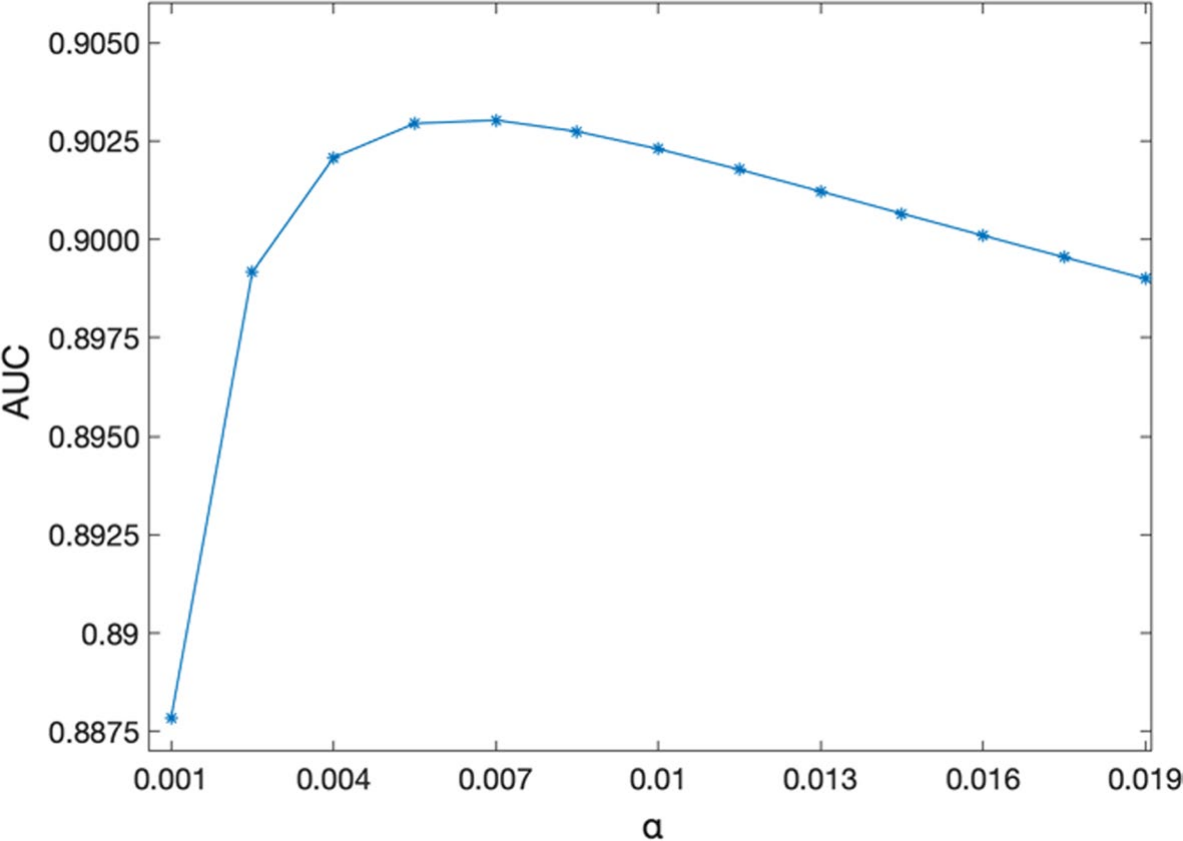
AUCs of different parameter α

In order to study the sensitivity of GKLOMLI to the parameter *α*, 13 different numbers for parameter α are used in the range of 0.001–0.019 (0.001, 0.0025, 0.004, …, 0.019) with step of 0.0015, when implementing 5-fold CV experiments. Based on these parameters setting, the results are plotted (see Fig. 3), in which the highest AUC of 0.9030 is obtained with *α* at 0.007. The distribution of AUC on a bell-shape curve indicates that it is easy to optimize the proposed model. Moreover, the distribution tends to reach the peak as *α* = 0.0055 and AUC = 0.9029. Thus, it demonstrates the robustness of the model to the setting of parameter.

## Discussion

Our proposed method proposed to transform the side information of RNAs into similarity and integrate that for further model training and prediction. The extra profiles of RNAs have been released constantly, which can be used for bioanalysis. In machine learning, fusing side information into former original data can offer more features of the entity information. To some extent, the integrated features can help train a model with better performance. The state-of-the-art methods are proposed to use different bio-information to improve the model performance. Specifically, LCBNI is based on sequence information. LNRLMI and EPLMI introduced expression profile of RNAs. INLMI integrated sequence information and expression profile. LMNLMI utilized bio-functional profile, sequence information and expression profile. The extra bio-information is introduced for improvement, but the results point out that more is not always better. However, the biological profiles of some specific RNAs haven’t been released, which leads to information loss. Such a problem largely results in low accuracy. In the pipeline of our proposed method, the integrated network completely relies on the network profile, which indicates its general application in many link prediction problems. The limited data of the interaction profile can be fully utilized by employing the Gaussian kernel-based method to transform them into a similarity matrix for information supplement. In further experiments, *k*-fold CV was implemented for performance evaluation. From the LOO-CV and *k*-fold CV experiments with parameter *k* at 2, 5, and 10, the results illustrated that more information offered can yield a higher AUC that means better performance. By integrating different biological profiles, the model based on the interaction profile yielded the highest AUC that indicated the Gaussian kernel-based method employed on a network profile is effective and reliable. Comparing the experiment results among the existing studies on miRNA–lncRNA interaction prediction, our proposed method performs well.

## Conclusions

The process of investigation of miRNA–lncRNA interactions still needs to make more effort for its essential role in potential regulation mechanisms in ceRNA network. In this paper, we proposed to employ the Gaussian kernel-based method on interaction profile to construct the network similarity and a linear optimization-based link prediction model. From all experiment results, it suggests that (1) ceRNA network contains the underlying information and can be extracted for network integration, and (2) information fusion is helpful for further model training in collaborative interactions between miRNAs and lncRNAs. Our proposed method can be a powerful tool to decipher the underlying regulation mechanisms in ceRNA network.

## Methods

### Dataset

In this work, there are 4 kinds of profiles used for investigating the interactions of miRNA–lncRNA from various databases, including miRNA–lncRNA interaction profile, sequence information, bio-functional profile, and expression profile. The interaction profile descripts the miRNAs interact with lncRNA or not, in which value of 1 denotes interaction, and value of 0 denotes non-interaction. Sequence information is that an RNA is a fix-length string of 4 kinds of bases including A, U, C and G. The bio-functional profile is arranged by a kind of gene annotation and interactions between RNAs and their targets. The expression profile is a series of numerical values that represent the expression level of a specific RNA in a cell line or tissues in vivo.

MLIs are obtained from the lncRNASNP database (released in Feb. 2017), was download at http://bioinfo.life.hust.edu.cn/lncRNASNP [18]. 8091 miRNA–lncRNA target pairs are provided from the database derived from 108 CLIP-Seq datasets, which are strongly related to bio-experimental studies. After processing data de-duplication, 5118 MLIs involving in 275 miRNAs and 780 lncRNAs are used for constructing the miRNA–lncRNA interaction network.

The sequence information is available in miRbase and LNCipedia databases at http://www.mirbase.org/ and https://lncipedia.org/, respectively [51, 52].

The bio-functional profiles of miRNAs are collected from miRTarBase of version 6.1 [53, 54]. 272 bio-functional profiles of miRNAs are collected. To obtain the functional profile of lncRNAs, the Lnc-GFP method was applied to learn the probable bio-function of lncRNAs from the coding-non-coding co-expression network.

The expression profiles of miRNAs and lncRNAs are downloaded from the microRNA.org database and the NONCODE database, respectively [19, 55]. 8 cell lines and 16 types of tissues are involved in the expression profiles of lncRNAs, as well as 172 dimensions of cell lines and tissues in the expression profiles of miRNAs. The numbers of expression profiles of miRNAs and lncRNAs are 230 and 450, respectively.

### Constructing RNA similarity for side information

In the pipeline of our proposed method, RNA similarities are regarded as the side information in the constructed observed miRNA–lncRNA interaction network, considering the observation that RNAs with similar clusters of targeting RNAs have more similarities in their profiles. Four kinds of similarities were constructed, including Gaussian kernel-based network similarity, sequence-based similarity, bio-function similarity, and expression profile-based similarity.

The Gaussian kernel is widely used in many fields for its efficiency in refining useful information from any input.

Given a profile $PF \in {\mathbb{R}}^{n \times d}$ with *n* RNA samples and *d* dimensions of the profile, the Gaussian kernel-based similarity value between the *i*-th and *j*-th sample is calculated as follow:

1 $$GKS\left( {r\left( i \right),r\left( j \right)} \right) = exp\left( { - \gamma_{r} PF\left( {r\left( i \right)} \right) - PF\left( {r\left( j \right)} \right)^{2} } \right),$$

where $\upgamma _{r}$ denotes the Gaussian kernel bandwidth. Its definition is as follow:

2 $$\upgamma _{r} = \left[ {\left( {\mathop \sum \limits_{i = 1}^{n} PF\left( {r\left( i \right)} \right)^{2} } \right)/n} \right]^{ - 1} .$$

To construct sequence-based similarity, a sequence alignment method was employed. The Needleman–Wunsch method is encapsulated in the *pairwise2* package of *Biopython* under the environment of Python. In the parameter setting, the identification score, gap-open penalty, and gap-open extending penalty were at 2, − 0.5, and < 0.1, respectively.

The function profile of RNA is related to so many entities. A set theory-based method was applied to construct the function similarity. The definition of the algorithm is as follow:

3 $$FS\left( {r_{a} ,r_{b} } \right) = \frac{{{\text{card}}\left( {RA\left( {r_{a} } \right) \cap RA\left( {r_{b} } \right)} \right)}}{{\sqrt {{\text{card}}\left( {RA\left( {r_{a} } \right)} \right)} \cdot \sqrt {{\text{card}}\left( {RA\left( {r_{b} } \right)} \right)} }}$$

where $r_{a}$ and $r_{b}$ denote two RNAs, and $RA\left( \cdot \right)$ denotes the functional annotations of RNA.

The Pearson correlation coefficient measure is one of the high-efficiency similarity measurements. The definition of it is as follow:

4 $${\text{PCCM}}\left( {r_{a} ,r_{b} } \right) = \frac{{\mathop \sum \nolimits_{i = 1}^{N} \left( {EX\left( {r_{a} ,i} \right) - \overline{{EX\left( {r_{a} } \right)}} } \right)\left( {EX\left( {r_{b} ,i} \right) - \overline{{EX\left( {r_{b} } \right)}} } \right)}}{{\sqrt {\mathop \sum \nolimits_{i = 1}^{N} \left( {EX\left( {r_{a} ,i} \right) - \overline{{EX\left( {r_{a} } \right)}} } \right)^{2} \mathop \sum \nolimits_{i = 1}^{N} \left( {EX\left( {r_{b} ,i} \right) - \overline{{EX\left( {r_{b} } \right)}} } \right)^{2} } }}$$

where $EX\left( {r_{a} ,i} \right)$ denotes the *i*-th value of *N* elements in the expression profile of RNA $r_{a}$ and $\overline{{EX\left( {r_{a} } \right)}}$ denotes the mean value of the expression profile of RNA $r_{a}$.

### A linear optimization-based method for inferring miRNA–lncRNA interactions

**Fig. 4:**
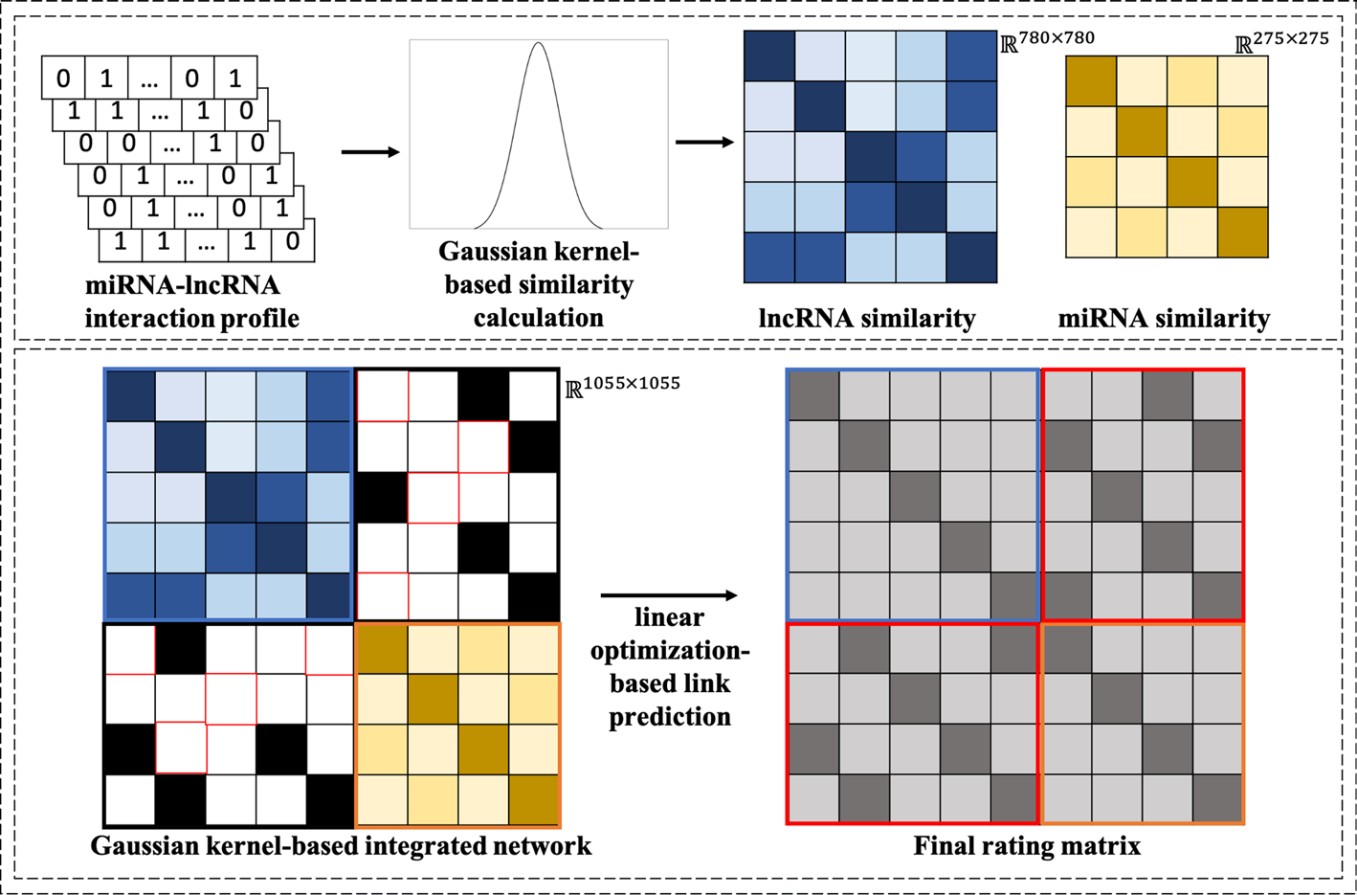
Flowchart of GKLOMLI model

In the pipeline of our proposed method, a semi-supervised learning algorithm is introduced to infer miRNA–lncRNA interactions from a constructed informative network that contains side information (see Fig. 4). In detail, in the framework of the proposed prediction model, we first integrate the given observed miRNA–lncRNA interaction network with the side information (bio-similarity) mentioned in the last section. Then, a linear optimization-based model is trained based on the integrated network. Notice that the similarities of miRNA and lncRNA are derived from the same kind of bio-profile.

Since more and more researches support the assumption that two RNAs with more similarities in profiles have a greater possibility of interacting with a common target RNA cluster, fusing the side information for training can be conducive to the investigation of miRNA–lncRNA interaction.

Given the similarity matrixes of miRNA and lncRNA, i.e., $Sim_{miRNA}$ and $Sim_{lncRNA}$, the integrated network is constructed with the adjacent matrix of miRNA–lncRNA interactions $Adj \in {\mathbb{R}}^{{n_{miRNA} \times n_{lncRNA} }}$ as follow:

5 $$Adj^{\prime} = \left[ {\begin{array}{*{20}c} {Sim_{miRNA} } & {\quad Adj} \\ {Adj^{\top} } & {\quad Sim_{lncRNA} } \\ \end{array} } \right]$$

where $Adj^{\prime}$ can be regarded as a weighted graph $G\left( {V,E,W} \right)$ with vertices *V*, edges *E*, and weighting *W.* Noted that $Adj^{\prime}$ is a real symmetric matrix, in which each row of a vertices in $Adj^{\prime}$ denotes a pathway regarded as edges with weights to other vertices. A target rating matrix related to $Adj^{\prime}$ and a contribution matrix *C* can be denoted as follow:

6 $$RS = Adj^{\prime} \cdot C$$

Each element $RS\left( {i,j} \right)$ can be represented by unfolding as follow:

7 $$RS\left( {i,j} \right) = \mathop \sum \limits_{k} Adj^{\prime} \left( {i,k} \right) \cdot C\left( {k,j} \right)$$

Then, we set a target function to get a final target matrix *RS* by solving it as a linear optimization problem. The definition is as follow:

8 $$\mathop {\min }\limits_{C} \alpha Adj^{\prime} - Adj^{\prime} \cdot C + C$$

where C should be with a small magnitude and $\alpha$ is a parameter for constraint. To solve the above equation, it can be treated as a minimum optimization problem by using the Frobenius-2 norm as follow:

9 $$\begin{aligned} F & = \alpha Adj^{\prime} - Adj^{\prime} \cdot C^{2} + C^{2} \\ & = \alpha {\text{Tr}}\left[ {\left( {Adj^{\prime} - Adj^{\prime} C} \right)^{\top} \left( {Adj^{\prime} - Adj^{\prime} C} \right)} \right] + Tr\left( {C^{\top} C} \right) \\ & = \alpha {\text{Tr}}\left( {Adj^{{^{\prime}\top}} Adj^{\prime} - Adj^{{^{\prime}\top}} Adj^{\prime} C - C^{\top} Adj^{\prime \top} Adj^{\prime} + C^{\top} Adj^{{^{\prime}\top}} Adj^{\prime} C} \right) + Tr\left( {C^{\top} C} \right) \\ \end{aligned}$$

10 $$\frac{\partial F}{{\partial C}} = \alpha \left( {2Adj^{{^{\prime}\top}} Adj^{\prime} C - 2Adj^{\prime \top} Adj^{\prime} } \right) + 2C$$

Let the above equation to be zero, C can be obtained:

11 $$C^{*} = \alpha \left( {\alpha Adj^{{^{\prime}\top}} Adj^{\prime} + E} \right)^{ - 1} Adj^{\prime \top} Adj^{\prime}$$

where *E* is the identity matrix related to $Adj^{\prime}$. The final rating matrix can be represented as follow:

12 $$RS = Adj^{\prime} C^{*}$$

## Data Availability

The data pertaining to the present study has been included in table and/or figure form in the present manuscript. And all datasets and computational code underlying this study are available in an online archive https://github.com/leon-lg-wong/GKLOMLI.
